# Colorectal Liver Metastases

**DOI:** 10.1155/2011/285840

**Published:** 2011-06-06

**Authors:** Ashraf J. Haddad, Murad Bani Hani, Timothy M. Pawlik, Steven C. Cunningham

**Affiliations:** ^1^Department of Surgery, Saint Agnes Hospital, Baltimore, MD 21229, USA; ^2^Department of Surgery, Johns Hopkins University, Baltimore, MD 21231, USA; ^3^Pancreatic and Hepatobiliary Surgery, Saint Agnes Hospital, 900 Caton Avenue, Mailbox no. 207, Baltimore, MD, 21229, USA

## Abstract

The diagnosis and management of CRLM is complex and requires a multidisciplinary team approach for optimal outcomes. Over the past several decades, the 5-year survival following resection of CRLM has increased and the criteria for resection have broadened substantially. Even patients with multiple, bilateral CRLM, previously thought unresectable, may now be candidates for resection. Two-stage hepatectomy, repeat curative-intent hepatectomy, and even selected resection of extrahepatic metastases have further increased the number of patients who may be treated with curative intent. Multiple liver-directed therapies exist to treat unresectable, incurable patients with adequate survival benefit and morbidity rates.

## 1. Introduction

In spite of the many advancements in molecular characterization, screening, diagnosis, surgical technique, and chemotherapeutics over the past several decades, colorectal cancer (CRC) remains a major health problem in the USA. It is the third most common cancer in males and females and is the second leading cause of cancer death [1]. In 2010, 142 570 new cases of CRC in the USA were estimated, and over a third of these patients will die of their disease [1], usually as a result of distant metastatic disease. 

About 65% of all patients with CRC develop distant metastasis, the liver being the most common site (40%) [2]. Colorectal liver metastasis (CRLM) may be diagnosed either synchronous or metachronous with the diagnosis of the primary tumor. In a recent French epidemiologic study, the proportion of patients who presented with synchronous and metachronous liver metastasis was equal: 14.5% of patients had synchronous CRLM while the rate of metachronous CRLM at 5 years was also 14.5% [3]. Of all patients with CRLM, approximately 25% will have metastasis confined to the liver and be resectable with curative intent [2, 4–6]. 

Due to therapeutic advances in the management of CRC, the case-fatality rate associated with CRC decreased by 10% (33% relative change) between 1990 and 2006 [1]. Data on the survival of CRC patients with liver metastasis, however, are not as defined, with 5-year survival ranging in the literature depending on a number of factors including patient selection: five-year survival rates for patients with synchronous and metachronous CRLM in a recent nonselected population series were 3.3% and 6.1%, respectively, [3]. In large (>200 patients) series of selected patients published in the 1990s comprised of patients who underwent curative resection of CRLM, overall 5-year survival was reported to range from 32 to 37% [7–9], and more recently, with the introduction of more effective cytotoxic chemotherapy agents in the 2000s, 5-year survival following resection of CRLM has been reported to range from 37 to 58%; [10–14] available 10-year survival rates in the literature range rather consistently between 22% and 28% (Table 1).

## 2. Prognostic Factors in CRLM

Many prognostic factors have been reported to be associated with risk of disease recurrence and survival following hepatic resection of CRLM. In 1997, Yasui et al. developed a macroscopic classification of liver metastasis, classifying liver metastasis into simple nodular (SN) lesions, which have a smooth and distinctive border, versus confluent nodular (CN) lesions, which are multinodular tumors with irregular borders [18]. Biological behavior and the degree of invasiveness were different between the two groups; vascular infiltration, lymph node metastasis, and invasion of adjacent viscera were all more common in the CN group versus the SN group. Five-year survival was 42% in the SN group but only 23% in the CN group [18].

Other factors subsequently associated with prognosis in patients with CRLM have included stage of the primary tumor, interval from primary to metastatic tumor diagnosis, number and size of metastases, status of surgical margins, presence of extrahepatic recurrence or metastasis, presence of satellite lesions, and serum levels of CEA, alkaline phosphatase, and albumin [9, 16, 17, 19]. Several groups have used some of these factors to create scoring systems based on large series of patients undergoing resection of CRLM, such as those by Nordlinger et al. [16], Fong et al. [9], Schindle et al. [17], and several others (Table 2). 

Some groups, such as Schindle et al., have applied the scoring system to patients before, as opposed to after, resection. Colon cancer stage, number of liver metastases, and serum levels of CEA, alkaline phosphatase, and albumin were identified as independent prognostic factors [17]. Based on these factors, Schindl et al. developed a scoring system that stratified patients into 3 groups with good, intermediate, and poor prognosis: although liver resection improved survival in all three groups, the 5-year survival of patients in the poor, moderate, and good prognosis groups were 0%, 20%, and 62% [17] (Table 2(c)). 

In addition, several groups have developed nomograms arguing that they prognosticate better than scoring systems [20–22]. Nathan et al. have recently compared the prognostic ability of several common scoring systems and suggested that conditional survival (CS) estimates (defined as the survival probability after a given length of survival) may provide improved prognostication, given that survival probabilities change over time [23]. For instance, review of an international database of 949 patients undergoing curative-intent resection of CRLM revealed a 5-year overall survival of 45% and a 10-year survival of 22%, but the 5-year CS (CS_5_) at 5 years (i.e., the probability of surviving another 5 years conditional on already having survived 5 years) was 50% [23].

## 3. Management of Patients with Resectable CRLM

### 3.1. Surgical Considerations Regarding Chemotherapy

**Figure 1:**
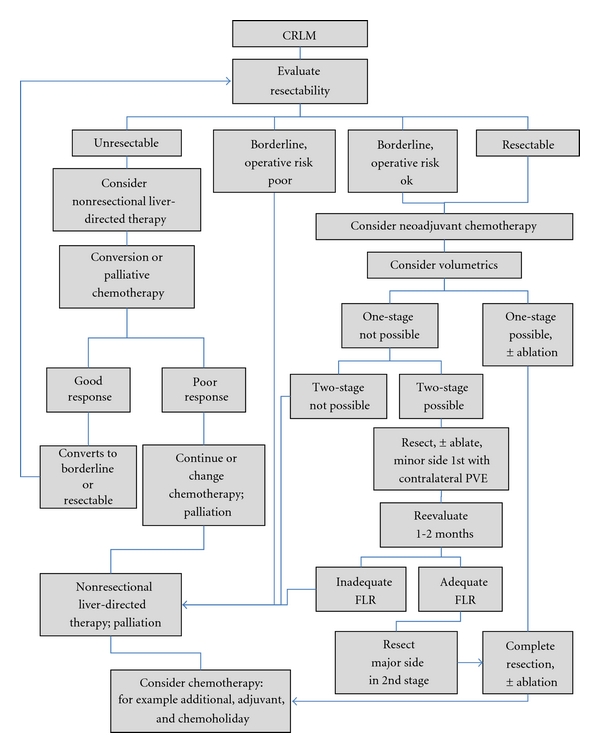
Simplified flow diagram of the management of CRLM. “Resectability” is defined in the text. See text for the abbreviations.

The use and timing of chemotherapy in patients with CRLM remains a subject of much debate and was recently the subject of an educational review series on controversies in the management of CRLM [24, 25]. Chemotherapy may be given prior (neoadjuvant) to the colectomy, between the colectomy and the hepatectomy, or after both the hepatectomy and the colectomy, which themselves may be staged or simultaneous. Few would disagree that a patient with a low-risk solitary metachronous CRLM should proceed directly to resection or that in cases of multiple, bilateral, synchronous CRLM, neoadjuvant chemotherapy is preferred. However, while some surgeons [25] argue that treatment of not only synchronous, but also most patients with CRLM should start with chemotherapy (Figure 1), others [24] have argued that the traditional paradigm of recommending neoadjuvant chemotherapy for resectable synchronous CRLM should be reconsidered, citing increased postoperative complications related to chemotherapy-induced hepatotoxicity, the rarity of a complete pathologic response to chemotherapy, and an inability to identify during a short course of neoadjuvant therapy those patients who may not benefit from resection, such as those with aggressive, occult, extrahepatic disease.

Several theoretical advantages of preoperative (both *neoadjuvant* for initially resectable and *conversion chemotherapy* for initially unresectable (Figure 1)) exist in cases of synchronous CRLM. These advantages include (1) selection: it may detect patients with occult, extrahepatic, chemoresistant metastases who could be spared an unhelpful operation, (2) assessment: it can test the responsiveness of the lesion to chemotherapy and guide postoperative chemotherapeutic drug choices, (3) systemic therapy: it can theoretically kill dormant or micrometastatic cells to increase the chance at total-body eradication, (4) downstaging: it may decrease the size of metastases and render more patients resectable [6] or the resectable tumors more easily resectable, sparing more normal parenchyma [26, 27], and (5) prognosis: response to chemotherapy may predict survival. Support for this last theoretical advantage has largely come from comparisons of patients with synchronous CRLM who either do or do not receive neoadjuvant chemotherapy [28], from series of metachronous-only cases [29], or from comparisons of mixed patients with synchronous and metachronous CRLM [30], which have suggested that response to neoadjuvant chemotherapy correlates with better overall survival. However, more recent data derived from comparisons of patients who all received neoadjuvant chemotherapy, and all of whom had synchronous disease showed no correlation between overall survival and response to neoadjuvant chemotherapy [31].

Surgery is the mainstay of management and the only chance for a cure for these patients, but recent level I data (the EORTC Intergroup trial 40983) have suggested a survival benefit associated with perioperative chemotherapy using FOLate, 5-Fluorouracil, and OXaliplatin (FOLFOX) [32]. The EORTC trial studied 364 patients from 78 hospitals and compared the progression-free survival in patients who received perioperative chemotherapy with that of patients who underwent resection alone. Six cycles of FOLFOX were administered before and after resection in patients with up to four liver lesions and histologically proven CRC. There was a statistically significant absolute increase in progression-free survival of 7.3% in randomized patients at 3 years but not in the intention-to-treat analysis [32]. Although perioperative chemotherapy is associated with longer survival than resection alone, and despite the numerous theoretical advantages associated with its use, there are significant disadvantages. These include delaying surgery, progression of chemotherapy-unresponsive CRLM from resectable to unresectable during neoadjuvant therapy, a higher rate of reversible surgical complications, chemotherapy-associated liver injuries, and, counterintuitively, excessive response of the metastases making them difficult to find at surgical exploration [24, 25]. 

Among these disadvantages, chemotherapy-associated hepatotoxicity likely receives the greatest attention from surgeons evaluating patients with CRLM [33]. At least two types of histologically identifiable injuries have been identified: fatty liver changes such as steatosis and steatohepatitis (associated with fluorouracil and irinotecan) and vascular sinusoidal injury (associated with oxaliplatin). Many retrospective studies have focused on the correlation between outcome (namely, complications) and chemotherapy-associated hepatotoxicity, some finding that hepatotoxicities such as steatosis [34] and steatohepatitis [35] independently predicted postoperative morbidity and mortality, respectively, and some finding no association between chemotherapy-associated hepatotoxicity and complications [36]. Results from the prospective EORTC trial, which randomized 364 patients into a surgery-only group and a perioperative chemotherapy group, revealing a small increase in the reversible postoperative complications in the chemotherapy group but no difference in mortality, likely best approximates the true effect [32].

Regarding the so-called “targeted” or “biologic” therapies, those aimed at vascular endothelial growth factor (VEGF) and epidermal growth factor receptor (EGFR) deserve mention. Bevacizumab (Avastin) is a monoclonal antibody that targets VEGF and has recently been shown to result in significantly improved survival of patients with metastatic colorectal cancer [37]. Similarly, the EGFR inhibitors cetuximab and panitumumab have recently shown activity in patients with CRLM whose tumors have nonmutated (wild-type) *KRAS* (reviewed in [38]). Due to increased risk of bleeding and impaired wound healing [38, 39], bevacizumab is typically withheld for 6–8 weeks prior to operation by most surgeons. The use of these targeted agents has not been associated with significant hepatotoxicity [40–42]. The effect of bevacizumab on liver regeneration, however, is less clear, given that some studies have found no significant impairment in regeneration after portal vein occlusion [40] while other data suggest that patients who are older than 60 year or who receive more than ≥6 cycles of bevacizumab had attenuated hypertrophy [43]. Some data have even suggested a protective effect on liver parenchyma exposed to cytotoxic chemotherapy, especially oxaliplatin-based regimens [44, 45], which is consistent with the observation that VEGF is implicated in vascular sinusoidal injury [46].

### 3.2. Staged versus Simultaneous Resection of the Primary CRC and the CRLM

In addition to the decision regarding the timing of chemotherapy relative to the hepatectomy, the planning of the colectomy relative to the hepatectomy requires careful consideration in cases of synchronous and resectable CRLM. These patients may undergo either a combined resection of the colon and liver disease or a staged resection of the primary CRC and the metastatic liver disease at two separate operations. Typically, the primary CRC is resected first, since it may cause current or imminent symptoms such as obstruction or bleeding. However, in select cases where the liver disease is marginally resectable and the primary CRC is small, the liver resection may be performed first to avoid progression of the CRLM to unresectability.

Procedure choice and order must be tailored to the individual patient, and few data exist to guide this decision. Surgeons should consider the complexity of both the hepatectomy and the colectomy, the ability to achieve adequate exposure with a single incision, the level of individual technical abilities, and the likelihood of progression of colonic symptoms or progression of the CRLM. Most of the literature available (Table 3) is retrospective and addresses predominantly the risk of morbidity and mortality associated with the two approaches. Two recent systematic reviews have summarized this literature. Hillingso and Wille-jorgensen included 16 articles and found that all contained significant bias, since the characteristics of the staged and the simultaneous groups were not usually equivalent: staged patients more often had left-sided colon primary tumors and larger, more numerous, and bilateral liver metastases, whereas patients with right-sided colon primaries and patients with small liver lesions in which a curative resection could be achieved with a minor resection were more likely to have the combined [61]. They recommended, on the basis of only level-II to -III evidence (grade C recommendation) that combined resections be performed in appropriately selected patients since this approach is associated with a shorter length of stay and less morbidity, with similar five-year survival [61]. A second systematic review by Chen et al., suffering from many of the same biases, evaluated 14 studies comprising 2,204 patients comparing simultaneous and staged resections and found that patients undergoing simultaneous resections had a similar mean operative time and blood loss and shorter length of stay and lower morbidity rate [62].

In general, small, especially left-sided, CRLM are easily accessible via the standard midline incisions used for the colectomy and may be safely combined with most colorectal resections [58]. Even complex resections such as lower anterior resections and abdominoperineal resections may be performed simultaneously with minor hepatectomies. Similarly, a straightforward right hemicolectomy may be combined with a larger hepatectomy. In some cases of multiple, bilateral, synchronous CRLM, a colon resection may be combined with one stage of a two-stage liver resection, as discussed below.

### 3.3. Multiple Bilateral Liver Metastases

The extent of disease amenable to curative-intent surgery (CIS) has increased in recent years. The now-historic teaching that the determinant of resectability in CRLM was a certain size or number of metastases fell by the wayside once it became clear that what matters most is not what is resected, but rather what is left behind, namely, negative margins and adequate functional liver parenchyma (>20% of a healthy liver) with preserved inflow, outflow, and bile drainage. Three main paradigm shifts in the treatment of bilateral CRLM have occurred to achieve resection of more extensive CRLM: (1) a trend toward parenchyma-sparing approaches over time, (2) increasing use of ablation and repeat CIS, and (3) the use of a 2-stage hepatectomy. 

Although not exclusive to cases of multiple, bilateral CRLM, the trend toward parenchyma-sparing approaches was exemplified at Memorial Sloan-Kettering Cancer Center (MSKCC) in a report of their experience in 440 patients undergoing 443 procedures over an 11-year period from 1992 to 2003 [63]. Of these patients, only 8.4% had synchronous lesions. An acceptable 29% rate of major complications and a 5.4% 90-day mortality rate were observed. Over time, the operative technique tended toward a decrease in major resections and an increase in smaller multiple resection and ablations, which correlated with a decrease in blood loss, hospital stay, and 90-day mortality, but no difference in disease-specific survival and liver recurrence [63]. 

**Figure 2:**
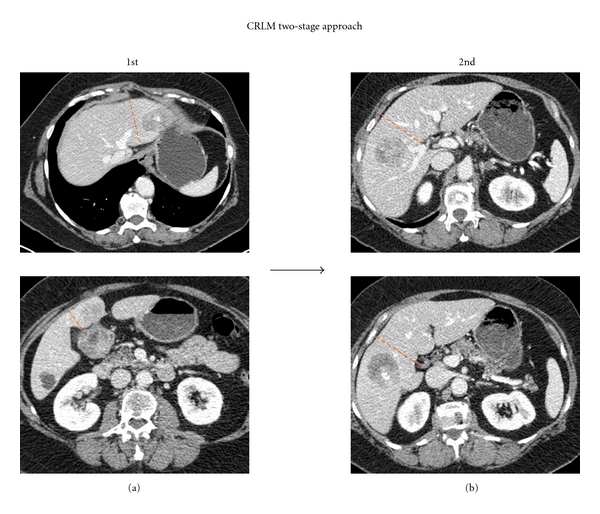
Example of patient with bilateral hepatic metastases managed with a two-stage hepatectomy approach. (a) During the first stage, the disease in the left hemiliver was resected. (b) During the subsequent second stage, a formal right hemihepatectomy was performed to extirpate the residual disease in the right hemiliver.

Between 20% and 30% of patients with CRLM (without extrahepatic disease) have such extensive bilateral liver disease; however, that complete extirpation at a single operation is not possible while maintaining an adequate future liver remnant (FLR) [64, 65]. These patients may be candidates for a 2-staged hepatectomy, in which a portion of the liver disease is removed and the contralateral portal vein is occluded, followed by a period of typically 1 to 3 months to allow hypertrophy of the remaining liver and a curative-intent, second-stage hepatectomy (Figures 1 and 2). Portal vein occlusion is typically performed either by intraoperative ligation (PVL) or subsequent percutaneous embolization and when adequate, induces significant hypertrophy, increasing the size of the FLR to decrease the risk of postoperative liver insufficiency.

Whether to undertake the minor or the major liver resection first remains debatable. Performing the major resection first was the approach taken in the seminal report by Adam et al., in which 16 patients underwent systemic chemotherapy followed by resection of the largest possible number of metastases in a first stage, followed by a second hepatectomy 4 months later. The mortality was 0%, and the morbidities for the first and second stages were 31% and 45%, with a 3-year survival of 35%. Although other groups have also opted for the major-first approach [66], most centers performing 2-stage hepatectomy perform the minor hepatectomy first [65]. The minor-first approach offers several advantages, including avoiding intraoperative manipulation of the FLR during the higher-risk second stage, improving selection of patients eligible for major hepatectomies since those with progressive disease may be spared a major procedure, and removing metastatic disease from the hemiliver to undergo hypertrophy, since theoretically (and observationally [67]) tumors in liver undergoing hypertrophy may grow faster than those in liver deprived of portal blood flow (although concomitant intraarterial chemotherapy may mitigate such growth [68]). When carefully applied to selected patients, 3-year survival ranging from 35% to 86% is achievable, comparable in some studies to survival following a planned one-stage resection [65, 69]. 

### 3.4. Recurrence after Curative-Intent Surgery

Patterns of recurrence following CIS have been studied in a recent, large, international, multi-institutional analysis of 1669 patients undergoing resection only (90%), resection plus ablation (8%), or ablation alone (2%) [70]. Within 2 years, most patients developed a recurrence, either intrahepatic only (43%), extrahepatic only (36%), or intra- and extrahepatic (21%) [70]. Patients selected to undergo resection combined with ablation as their initial operation had a median of 6 metastases; recurrence, but not survival, was significantly associated with the number of lesions ablated [71]. Among all patients who developed an intrahepatic recurrence, irrespective of whether the initial CIS was resection, ablation, or both, nearly 40% were candidates for repeat CIS, and a small subset underwent a third and fourth CIS with similarly low morbidity and mortality [72], a trend that begins to render CRLM akin to a chronic disease. With subsequent CIS [72], the extent of hepatic resection not surprisingly decreased significantly and RFA was more frequently used (>20%, cf. <10% in the first CIS [70]). The five-year survival following the first, second, and third CIS was 47.1%, 32.6%, and 23.8%, respectively, [72].

## 4. Management of Patients with an Initially Unresectable CRLM

### 4.1. Chemotherapy versus Resection of the Primary CRC in Patients with Unresectable CRLM

For the approximately 75% of patients with CRLM who are unresectable even by 2-stage hepatectomy, several treatment strategies exist. Criteria for unresectability of CRLM are typically considered to be major liver vascular involvement (e.g., of all 3 hepatic veins, the portal vein bifurcation, or the retrohepatic vena cava), bilateral dissemination requiring liver resection that would leave an inadequate FLR, and multiorgan or unresectable uniorgan extrahepatic disease [8, 24, 68, 73–75]. 

The best treatment strategy for unresectable CRLM with synchronous asymptomatic colorectal cancer is debatable [76, 77]. Two treatment options debated are CRC resection followed by chemotherapy or chemotherapy followed by colon resection only if the patient develops complications of the CRC or the CRLM is downstaged to resectability (see below). Throughout the 1990s, approximately 66% [78] to 72% [79] of patients with unresectable synchronous CRLM underwent resection of the primary CRC, although this predominant approach has been recently challenged, given recent improvements in systemic chemotherapy [74, 79, 80]. 

Advocates of resecting the bowel cancer first cite advantages of precise definition of nodal and peritoneal status, prevention of local complications of progression, a theoretical advantage of a reduction of total-body neoplastic mass, psychological benefit for the patient, and data showing a survival advantage [75, 76, 78, 81]. Proponents of the chemotherapy-alone or chemotherapy-first approach cite advantages of avoidance of postoperative mortality and morbidity, immediate treatment of the primary and metastatic disease with the potential to downstage unresectable CRLM to resectability, a low frequency of complications from unresected tumors, and data showing equivalent survival benefits [24, 74, 80].

In an effort to better stratify patients with unresectable CRLM according to risk of postoperative death, Vibert et al. performed a multivariate analysis that identified age >75 years and liver cytolysis (AST > IU/L) as criteria that served as independent predictors of early postoperative death in patients who, they argued, should therefore not undergo CRC resection: the 30-day postoperative mortality rate was 15% with neither criterion, 44% with one criterion, and 100% when both criteria were met [74].

In symptomatic patients, the decision for initial therapy is more straightforward and depends predominantly on the operative risk of the patient: endoscopic stents for high-risk patients and palliative resection in low-risk patients. In fact, among patients who receive initial chemotherapy with modern combination regimens, the vast majority of patients never require palliation of their primary tumor. Poultsides et al. evaluated 233 consecutive such patients with synchronous CRLM and an unresected primary CRC who received initial triple-drug regimens (oxaliplatin- or irinotecan-based) and found that only 7% required emergency operations and only 4% required nonoperative palliation (e.g., stenting) for complications of the primary CRC [82].

### 4.2. Downstaging from Unresectable to Resectable CRLM with Systemic Chemotherapy

As discussed above, a major advantage of initial chemotherapy in patients with unresectable CRLM is the immediate treatment of the liver metastases and the possibility to downstage them to resectability (Figure 1). In a retrospective series of nearly 1500 patients with CRLM, Adam et al. found that among 1104 unresectable patients treated with systemic chemotherapy, 138 (12.5%) had a response sufficiently robust to allow for curative-intent resection, with an overall 5-year survival of 33% [6]. 

Similarly, Nuzzo et al. compared 60 initially resectable patients with 42 initially unresectable patients receiving irinotecan-based chemotherapy and found that 15 (35.7%) of the latter group were converted from unresectable to resectable CRLM [83]. Operative complications, margin status, and 3-year overall survival (71% and 73%, resp.) were similar in both groups, although 3-year disease-free survival was higher in the primarily resectable patients (58%) than in the primarily unresectable but downstaged patients (31%); recurrence rates were 28% and 53%, respectively, and half of those recurrences in the latter group were reresected with median survival ranging from 9 to 67 months [83].

Selzner et al. have studied the combination of PVL and intraarterial chemotherapy to downstage unresectable CRLM with acceptable morbidity [68]. Of 11 patients included in this very small study, 6 had a radiographic response to chemotherapy and 4 were sufficiently downstaged to allow curative-intent resection; 2 of these died at 20 months and 2 were alive at 26 and 40 months. Unlike other studies not using intraarterial chemotherapy in combination with portal vein occlusion [67], this small study found that the growth rate of liver metastases in the regenerating hemiliver was not accelerated, despite parenchymal regeneration [68].

### 4.3. Nonoperative Liver-Directed Therapy

There are three broad categories of nonoperative liver-directed therapy: transarterial therapies, ablative therapies, and radiotherapies. All three are often reserved for cases in which a complete resection is not possible, either due to patient or tumor prohibitive factors. Overlap exists among these approaches and between them and system chemotherapy, with multiple approaches available for use in an individual patient.

### 4.4. Transarterial Therapies

Transarterial therapies include bland (simple thrombotic) transarterial embolization (TAE) alone, transarterial chemoembolization (TACE), continuous infusion via hepatic arterial infusion (HAI) pumps, isolated hepatic perfusion (IHP), and intraarterial radiotherapy (IART). Because liver tumors typically receive predominantly arterial and not portal blood flow [84, 85] and because one of the greatest disadvantages of systemic chemotherapy is systemic toxicity, regional transarterial chemotherapy offers an attractive treatment option for unresectable CRLM. TAE and TACE have been directly compared in small trials with similar median survival results (8–12 months) [86, 87]. Given the theoretical advantage of adding a chemotherapeutic agent (namely, the possibility of additive or synergistic effect of cytotoxicity from chemotherapy and ischemia from embolization), TACE is more commonly used than TAE. A larger, more recent series of 245 TACE treatments performed in 121 patients reported a 27-month overall median survival (from the development of the CRLM) using chemoembolization with cisplatin, doxorubicin, and mitomycin C as the chemotherapeutic agents [88]. Because TACE is predominantly studied in the treatment of hepatocellular carcinoma, its role in CRLM remains to be better elucidated.

HAI was first studied as sole treatment in the treatment of unresectable CRLM and has been compared to systemic chemotherapy alone, with most studies showing higher response rates with HAI, but not necessarily higher survival rates (ranging from 12–20 months) [89, 90]. Subsequently, HAI in combination with systemic chemotherapy has been found to achieve a response rate of 88% and median survival of 36 months [91]. HAI, however, has several disadvantages, which are made largely moot by the fact that it is performed in very few centers and therefore not available to the vast majority of patients with unresectable CRLM.

IHP was first reported in 1961 [92] and has since been studied using both operative and percutaneous approaches in a variety of patient populations with liver metastases [93]. Alexander et al. recently reported on factors associated with outcome in 120 patients from 1994 to 2004 at the National Cancer Institute with unresectable CRLM (median 8 metastases) who underwent IHP with either melphalan, tumor necrosis factor, or both [94]. A radiographic response rate of 61% and a median overall survival of 17 months were observed, but only the melphalan groups had significant association with radiographic response. Post-IHP HAI was performed in 38% of patients and was independently associated with improved survival [94]. In a European case-control study of patients with unresectable CRLM (median number not reported) from the same time period, IHP with melphalan (*N* = 99) was compared with systemic chemotherapy (*N* = 111) using capecitabine, irinotecan, and oxaliplatin and no difference in overall survival was detected [95]. Because the IHP technique and melphalan doses used in these studied differed, comparison is difficult, but the ability to deliver high doses of chemotherapy to the cancer-burdered organ with limited systemic toxicity is certainly attractive.

IART is the delivery of radiotherapy, typically Yttrium-90 (the only currently FDA-approved intraarterial therapy), a high-energy beta-particle-emitting radioisotope incorporated onto glass or resin microspheres, into the hepatic arterial system. Sharma et al. in a 2007 phase-I study compared ytrrium-90 IART with FOLFOX for unresectable CRLM (*N* = 20). Radiographic responses were observed in 90% of patients and stable disease in 10%; median progression-free survival was 9.3 months [96]. A recent Cochrane study [97] found a single randomized study comparing IART plus systemic chemotherapy versus systemic chemotherapy alone [98], which found a significant improvement in progression-free survival associated with IART, with no difference in quality of life.

### 4.5. Ablative Therapies and Radiotherapies

Ablation and external beam radiation may both be used in the palliative treatment of unresectable CRLM [99], although these modalities have a less prominent role than transarterial therapies.

## 5. CRLM in the Presence of Extrahepatic Disease

The presence of extrahepatic disease (EHD) in cases of CRLM presents a unique challenge to the patient and the surgeon. Although traditionally considered a contraindication to surgical treatment, resection of EHD is appropriate in highly selected patients. In a recent series of 1369 patients undergoing resection of CRLM from 1992 to 2007 at MSKCC, 127 underwent concomitant resection of EHD located in the lung (27%), portal lymph nodes (21%), adjacent structures locally invaded, including the diaphragm, portal vein, vena cava, or right adrenal/kidney (16%), “other” single-site metastases, including ovary, retroperitoneal lymph nodes, colorectal recurrence, subcutaneous tissue, and mediastinal lymph nodes (16%), the peritoneum (12%), and multiple sites (8%) [100]; 5-year survival was 26% and 49% in those patients with and without EHD, respectively. In both the thoracic [101] and the general surgical oncology [102] literature, the 5-year survival after resection of pulmonary and hepatic colorectal liver metastasis ranges between 30% and 60% in highly selected patients. Ideal patients to consider for resection are young, fit, with completely resectable hepatic disease, a single site of resectable EHD, and demonstrated responsiveness to systemic chemotherapy.

## 6. Summary

The diagnosis and management of CRLM is complex and requires a multidisciplinary team approach for optimal outcomes. Over the past several decades, the 5-year survival following resection of CRLM has increased and the criteria for resection have broadened substantially. Even patients with multiple, bilateral CRLM, previously thought unresectable, may now be candidates for resection. Two-stage hepatectomy, repeat curative-intent hepatectomy, and even selected resection of extrahepatic metastases have further increased the number of patients who may be treated with curative intent. Multiple liver-directed therapies exist to treat unresectable, incurable patients with adequate survival benefit and morbidity rates.

## Figures and Tables

**Table 1:** Selected Large (>200 patients) studies of resection for CRLM with 5- and 10-year survival.

		Survival		
First author [ref]	Year	*N*	5-Year	10-Year
Gayowski [7]	1994	204	32%	NR
Scheele [8]	1995	350	39%	24%
Fong [9]	1999	1001	37%	22%
Choti [10]	2002	226	40%	26%
Abdalla [11]	2004	348*	58%	NR
Pawlik [12]	2005	557	58%	NR
Wei [13]	2006	423	47%	28%
Tomlinson [14]	2007	612	37%	24%
Fortner [15]	2009	293	35%	24%

NR: not reported.

*Includes 190 patients undergoing resection only, 101 resection and ablation, and 57 ablation only.

**Table:** (a) Nordlinger prognostic system

Risk	*N*	No. of risk factors	2-year survival
Low	305	0–2	79%
Intermed	738	3-4	60%
High	230	5–7	43%

Risk factors are (1) age > 60 years, (2) extension of primary CRC into serosa, (3) node-positive primary CRC, (4) time interval from primary CRC to CRLM < 2 years, (5) size of largest CRLM ≥ 5 cm, (6) number of CRLM ≥ 4, (7) margin less than 1 cm [16].

**Table:** (b) Fong prognostic system

Clinical risk score for tumor recurrence						
	Survival					
	%					
Risk factors	1 yr	2 yr	3 yr	4 yr	5 yr	Median (months)

0	93	79	72	60	60	74
1	91	76	66	54	44	51
2	89	73	60	51	40	47
3	86	67	42	25	20	33
4	70	45	38	29	25	20
5	71	45	27	14	14	22

Risk factors are (1) node-positive primary CRC, (2) time interval from primary CRC to CRLM < 1 year, (3) number of CRLM > 1, (4) size of largest CRLM > 5 cm, (5) CEA > 200 ng/mL (reproduced from [9]).

**Table:** (c) Schindl prognostic system

Risk	*N*	Prognostic score	Median survival (months)	5-year survival (%)
Good	33	0–10	36	62
Mod	172	11–25	34	20
Poor	65	>25	11	0

Prognostic score = [(4 × Dukecode) + (6 × Metcode3) + (6 × *ln*Alkphos) + (2 × *ln*CEA) − Albumin] + 22, where Dukecode indicates Dukes stage A/B (score, 0) or C (score, 1), Metcode3, 1 to 3 metastases (score, 0) or more than 3 metastases (score, 1), *ln*Alkphos, natural logarithmic function of the serum concentration of alkaline phosphatase (U/L), *ln*CEA, natural logarithmic function of the serum concentration of CEA (*μ*g/L), and Albumin, the serum concentration of albumin (g/dL) [17].

**Table 3:** Staged versus simultaneous resection: 5-year survival and morbidity.

First author [ref]	Year	Design	Simultaneous				Staged			
			N	Age	5YS	Morbidity	N	Age	5YS	Morbidity
Vogt [47]	1991	Retro	19	NR	39%	5.3%	17	NR	0%	18%
Jaeck [48]	1999	Retro	28	56	NR	18%	31	60	NR	16%
Martin [49]	2003	Retro	134	64	NR	49%	106	61	NR	67%
Weber [50]	2003	Retro	35	58	21%	23%	62	60	22%	32%
Tanaka [51]	2004	Retro	39	64	53%	28%	37	65	47%	16%
Chua [52]	2004	Retro	64	63	29%	53%	32	61	43%	41%
Thelen [53]	2007	Retro	40	60	53%	18%	179	60	39%	25%
Turrini [54]	2007	Retro	57	60	32%	21%	62	59	25%	31%
Yan [55]	2007	Retro	73	60	36%	32%	30	59	37%	43%
Capussotti [56]	2007	Retro	70	65	31%	NR	57	60	32%	NR
Vassiliou [57]	2007	Retro	25	63	28%	72%	78	61	31%	76%
Reddy [58]	2007	Retro	135	57	NR	44%	475	58	NR	27%
Slupski [59]	2009	Retro	28	59	45%	14%	61	60	38%	13%
Martin [60]	2009	Retro	70	58	NR	56%	160	61	NR	55%

Retro: retrospective; 5YS: 5-year survival. “Simultaneous” and “Staged” refer to resection of the primary CRC tumor and the CRLM.
